# Screening for duplications, deletions and a common intronic mutation detects 35% of second mutations in patients with *USH2A* monoallelic mutations on Sanger sequencing

**DOI:** 10.1186/1750-1172-8-122

**Published:** 2013-08-08

**Authors:** Heather B Steele-Stallard, Polona Le Quesne Stabej, Eva Lenassi, Linda M Luxon, Mireille Claustres, Anne-Francoise Roux, Andrew R Webster, Maria Bitner-Glindzicz

**Affiliations:** 1 UCL Institute of Child Health, London, UK; 2 UCL Institute of Ophthalmology, London, UK; 3 Moorfields Eye Hospital, London, UK; 4 Audiovestibular Medicine, National Hospital for Neurology and Neurosurgery, London, UK; 5 UCL Ear Institute, London, UK; 6 CHU Montpellier, Laboratoire de Génétique Moléculaire, Montpellier, F-34000, France; 7 Inserm, U827, Montpellier F-34000, France; 8 Great Ormond Street Hospital, London, UK

**Keywords:** Usher syndrome, *USH2A*, Deletion, Duplication, Pseudoexon, Multiplex ligation dependant probe amplification (MLPA), Array CGH

## Abstract

**Background:**

Usher Syndrome is the leading cause of inherited deaf-blindness. It is divided into three subtypes, of which the most common is Usher type 2, and the *USH2A* gene accounts for 75-80% of cases. Despite recent sequencing strategies, in our cohort a significant proportion of individuals with Usher type 2 have just one heterozygous disease-causing mutation in *USH2A*, or no convincing disease-causing mutations across nine Usher genes. The purpose of this study was to improve the molecular diagnosis in these families by screening *USH2A* for duplications, heterozygous deletions and a common pathogenic deep intronic variant *USH2A*: c.7595-2144A>G.

**Methods:**

Forty-nine Usher type 2 or atypical Usher families who had missing mutations (mono-allelic *USH2A* or no mutations following Sanger sequencing of nine Usher genes) were screened for duplications/deletions using the *USH2A* SALSA MLPA reagent kit (MRC-Holland). Identification of *USH2A*: c.7595-2144A>G was achieved by Sanger sequencing. Mutations were confirmed by a combination of reverse transcription PCR using RNA extracted from nasal epithelial cells or fibroblasts, and by array comparative genomic hybridisation with sequencing across the genomic breakpoints.

**Results:**

Eight mutations were identified in 23 Usher type 2 families (35%) with one previously identified heterozygous disease-causing mutation in *USH2A*. These consisted of five heterozygous deletions, one duplication, and two heterozygous instances of the pathogenic variant *USH2A*: c.7595-2144A>G. No variants were found in the 15 Usher type 2 families with no previously identified disease-causing mutations. In 11 atypical families, none of whom had any previously identified convincing disease-causing mutations, the mutation *USH2A*: c.7595-2144A>G was identified in a heterozygous state in one family. All five deletions and the heterozygous duplication we report here are novel. This is the first time that a duplication in *USH2A* has been reported as a cause of Usher syndrome.

**Conclusions:**

We found that 8 of 23 (35%) of ‘missing’ mutations in Usher type 2 probands with only a single heterozygous *USH2A* mutation detected with Sanger sequencing could be attributed to deletions, duplications or a pathogenic deep intronic variant. Future mutation detection strategies and genetic counselling will need to take into account the prevalence of these types of mutations in order to provide a more comprehensive diagnostic service.

## Background

Usher Syndrome is the leading cause of inherited deaf-blindness, accounting for 50% of cases. The disorder is clinically and genetically heterogeneous and is divided into three clinical subtypes, Usher type 1 (USH1), Usher type 2 (USH2) and Usher type 3 (USH3). Classification between subtypes is based upon type of hearing loss and presence or absence of vestibular dysfunction [1,2]. In type 1, affected people have profound congenital deafness, absent vestibular function and prepubertal onset of retinitis pigmentosa (RP); in type 2 the hearing loss is moderate to profound (sloping pattern) congenital hearing loss, with normal vestibular function and pre or post-pubertal onset RP; and in type 3, hearing loss maybe pre- or post-lingual but progressive in course, with normal or abnormal vestibular function and often post-pubertal onset of RP. Individuals who do not have the usual phenotypes for each of these three subtypes are classed as atypical. Usher Syndrome is an autosomal recessive disorder. Previous studies have identified 12 loci and 10 causative genes. The most recently identified gene is *CIB2*, which is a rare cause of Usher Type 1 [3]. In addition to these 10 genes, *PDZD7* has been implicated as both a modifier and a potential contributor to digenic inheritance [4].

The prevalence of Usher Syndrome has been estimated to be 3–6 per 100,000, although recently it has been re-estimated to have a much higher frequency of 1 in 6,000 [5]. Usher type 2 is the most prevalent form accounting for more than half of reported cases. There are three genes underlying USH2: *USH2A, GPR98* and *DFNB31* (*WHRN)*, with *USH2A* accounting for 75-80% of cases [6-8]. The mutational spectrum of *USH2A* is diverse and includes nonsense, frameshift, missense and splice-affecting mutations, as well as deletions and small duplications [9]. Identifying the correct disease-causing variant is often confounded by the polymorphic nature of this gene, and the high frequency of novel mutations associated with this syndrome. For this reason, missense variants are assigned a value for their likelihood of causing disease ranging from Unclassified Variant 1 to 4 (UV1 to UV4). This classification system is based on frequency in controls, if the variant is novel in Usher syndrome, segregation with disease, and bioinformatic analysis of pathogenicity and conservation. UV4 variants are considered likely pathogenic, UV3 possibly pathogenic, UV2 possibly polymorphism, UV1 likely polymorphism [7,10]. Details of mutations and their ranked pathogenicity are recorded and revised in the Usher syndrome database, an invaluable tool in the molecular diagnosis of this disorder [9,10].

Despite recent sequencing strategies that have analysed nine Usher Syndrome genes, and other studies involving thorough sequencing of *USH2A*, 8-19% of USH2 individuals have just one heterozygous likely disease-causing mutation in *USH2A*[6-8], and 13% of USH2 patients have no convincing disease-causing mutations [7]. Unidentified mutations in USH2 individuals could lie in the promoter, regulatory regions, and deep intronic areas, all of which are not usually analysed during conventional mutation screening. Recently there has been a report of a pathogenic deep intronic variant *USH2A*: c.7595-2144A>G that causes the inclusion of a 152bp pseudoexon in the mRNA transcript, leading to the frameshift p.(Lys2532Thrfs*56) [11]. This was identified using investigation of RNA transcripts, underpinned by less invasive techniques for obtaining Usher gene RNA from affected individuals [12,13].

Missing variants might also be attributable to heterozygous deletions and duplications, either those involving single or multiple exons, or the whole gene. Current investigations into the genetic basis of Usher syndrome have focused on Sanger sequencing to detect mutations [7,8]. It is not possible to robustly detect deletions and duplications by sequencing alone, as this method is not sensitive to relative changes in the copy number of exons. Homozygous whole single exon or multi-exon deletions can be inferred from consistent PCR non-amplification, but these are not very common, especially in the absence of consanguinity. Large duplications and heterozygous large exonic deletions are in effect ‘invisible’ if the breakpoints are outside of the amplified region. Previous work has identified such deletions through haplotype analysis, but to be comprehensive this requires the individual to carry informative variants in every exon. More recent methodologies have looked for deletions and duplications using array comparative genomic hybridization (array CGH) and multiplex ligation dependent probe amplification (MLPA) [10,14,15]. These methods have not yet been utilised to detect deletions and duplications in *USH2A* in USH2 and atypical individuals. As this gene is the major genetic contributor to USH2, and occasionally a cause of atypical Usher, it is important to screen for deletions and duplications in *USH2A*.

In this study we aimed to improve the molecular diagnosis of USH2 and atypical Usher by searching for deletions and duplications in *USH2A* by MLPA and array CGH, and by screening for the pathogenic deep intronic variant *USH2A*: c.7595-2144A>G. We also sought to develop methods to analyse splicing variants at the RNA level.

We identified 35% of missing mutations in USH2 families with one previously identified (monoallelic) pathogenic/UV4/UV3 mutation in *USH2A*. These variants include five novel deletions in *USH2A* and one novel duplication. Results were confirmed by array CGH, and where possible by RNA extracted from nasal epithelial cells and dermal fibroblasts. This is the first time that proband derived fibroblasts have been used for the study of splicing variants in Usher Syndrome.

## Methods

### Patient and control DNAs

Thirty-eight USH2 and 11 atypical families were included in this study. Twenty-three of these USH2 families had one pathogenic/UV4/UV3 mutation in *USH2A.* The remaining 15 USH2 families and 11 atypical families had no convincing disease-causing mutations (no mutations above UV2/UV1) in nine Usher genes, *MYO7A, USH1C, CDH23, PCDH15, SANS, USH2A, DFNB31 (WHRN), GPR98*, or *CLRN1* (only *CIB2* not analysed as *CIB2* was described after the completion of this study). These probands were selected from 121 USH2 and 11 atypical families that were part of a previous mutation screening programme, the National Collaborative Usher Study [7]. For genotypes of the forty-nine families screened see Additional file 1. Clinical data for all atypical families, and USH2 families with mutations identified in this study, is given in Additional file 2. Informed consent was obtained from all participants. For nasal epithelial brushings and skin punch biopsies, additional informed consent for these procedures was given. Control DNA and RNA were obtained from consenting unrelated healthy individuals. This study adhered to the provisions of the declaration of Helsinki, and was approved by the National Research Ethics Committee - London South East.

### Multiplex ligation dependent amplification (MLPA)

The SALSA MLPA FAM labelled reagent kit with probe mixes P361-A1/ P362-A2 developed by MRC-Holland (MRC-Holland, Amsterdam, Netherlands), was used to detect deletions and duplications in the *USH2A* gene. Two MLPA probe mixes were required to encompass all 72 exons. Each probe mix contained 15 internal control probes; nine probes detected non-Usher genes on autosomes, four Q-oligonucleotides detected low DNA quantity and two D-oligonucleotides detected incomplete DNA denaturation. In addition to the 49 USH2 and atypical families, parents in two further families were included to act as positive controls and confirm the validity of the method. These families were previously identified to have large homozygous deletions in *USH2A*[7]. Family 221 had a homozygous deletion of exon 47, and family 683 had a homozygous deletion of exon 50–58. The latter of these was previously published as a homozygous deletion of *USH2A* exons 50–55. MLPA analysis however showed this was in fact a deletion spanning exons 50–58, which was homozygous in the proband and heterozygous in both parents. One deletion control and one healthy control were run per 10 proband DNA samples. Controls without DNA, consisting of TE buffer were used to check for contamination in reagents.

Reactions were performed as per manufacturer’s instructions [16]. Fragment size separation was conducted on the Applied Biosystems Inc. (ABI) 3730 DNA analyser with POP7 polymer capillary electrophoresis. Peak patterns were first evaluated using the raw data check list and the peak pattern evaluation flow chart available from MRC Holland (supplied as part of the MLPA general protocol). Data that passed peak pattern evaluation was normalised against negative controls, and dosage quotients for each probe calculated using Gene Marker v2.2.0 MLPA analysis software (SoftGenetics, Pennsylvania, USA) [17]. A probe dosage quotient value of less than 0.8 was considered a deletion, 0.8 to 1.2 normal, greater than 1.2 a duplication. Samples failed analysis if three or more of the nine supplied non-Usher gene control probes were deleted/duplicated.

### CGH array

A custom designed CGH-microarray chip (12 × 135 k) that includes 16 sensorineural hearing loss genes was used on a high-resolution microarray platform according to the manufacturer’s recommendations (Nimblegen; Roche Diagnostics, Basel, Switzerland). The CGH-microarray chip includes 77,366 probes covering the Usher-related genes (*MYO7A*, *CDH23*, *PCDH15*, *USH1C*, *USH1G*, *USH2A*, *GPR98*, *DFNB31*, *PDZD7*, *USH3A*) and their 10,000-bp 5’ and 3’ regions. The average probe length is 60 bases and the spacing between starts of probes covering exons and introns is 35 bp. The slides were scanned using InnoScan 900 A (Inopsys, Toulouse, France) and analyzed using Deva 1.2.1 software (Roche NimbleGen, Inc.).

### Deletion breakpoint mapping on genomic DNA

Primers were designed to amplify across deletion breakpoints, based on array CGH results. PCR and sequencing was conducted with Biotaq DNA polymerase (Bioline, London, UK) and Big Dye terminator v1.1 (Applied Biosystems, Texas, USA). Primer sequences are available on request.

### Nasal epithelial brushings

The lateral inferior turbinate was gently brushed five times with a 3 mm by 1.8 mm bronchial cytology brush (Diagmed Ltd, North Yorkshire, UK). Brushes were immediately placed in cell lysis buffer (supplied with NucleoSpin RNA II, Macherey-Nagel, Duren, Germany).

### Skin biopsies and fibroblast cell culture

Punch biopsies were taken from consenting patients who carried *USH2A*: c.7595-2144A>G. Biopsied skin was placed in medium (DMEM glutamax + with 10% fetal bovine serum, 80 Units/ml of Penicillin and 80 mg/L of Streptomycin). Fibroblasts were isolated and cultured according to standard procedures. The established fibroblast cell lines were trypsinised and passaged weekly as per standard protocols. For RNA extraction cells in the log phase of growth were pelleted and resuspended in cell lysis buffer (supplied with NucleoSpin RNA II, Macherey-Nagel, Duren, Germany).

### RNA extraction and cDNA synthesis

RNA was extracted from fibroblast cell pellets and nasal epithelial cells with NucleoSpin RNA II (Macherey-Nagel, Duren, Germany) as per manufacturer’s instructions. RNA was quantified and assessed for purity using Nanodrop 1000. For cDNA synthesis 2 μg of RNA was used for fibroblast samples, and 0.8 μg to 1 μg was used for nasal samples. cDNA was synthesised using the Bioline cDNA synthesis kit.

### Reverse transcription PCR (RT-PCR)

Genomic contamination was assessed in cDNA using intron-spanning primers for *GAPDH*. In addition to this all RT-PCR primers were designed to be intron-spanning. When necessary PCR reactions were nested with internal primers. Primer sequences are available on request.

### Accession numbers

Variants are described as per Human Genome Variation Society’s recommendations, where +1 is the A in the ATG translation start codon of *USH2A* accession number NM_206933.2.

## Results

### Identification of large genomic rearrangements in the *USH2A* gene

Forty-nine families without convincing biallelic disease-causing variants were screened for deletions and duplications in *USH2A* by MLPA. This group of families consisted of 23 USH2 families with one previously published pathogenic/UV4/UV3 mutation in *USH2A*, and 15 USH2 and 11 atypical families with only UV2/UV1, or no variants, across 9 Usher genes (i.e. no convincing disease-causing variants). For genotypes of all these families see Additional file 1.

**Figure 1:**
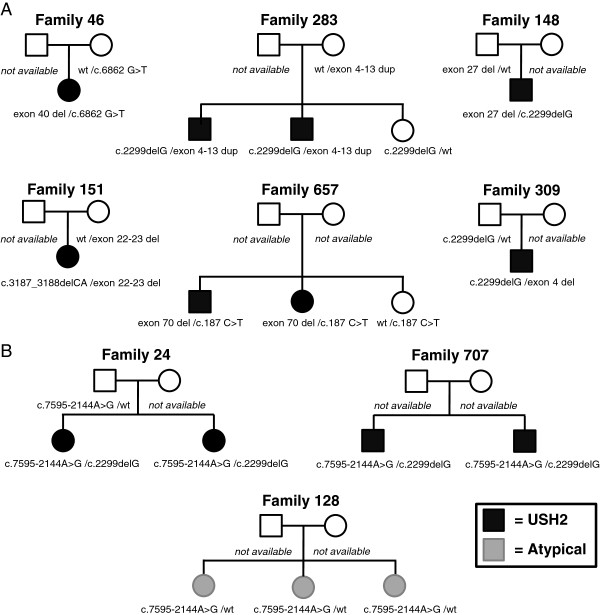
**Segregation analysis of families identified with *USH2A *deletions/duplications and *USH2A*: c.7595-2144A>G. A**. Six families were found to have deletions/duplications that segregated *in trans* with a previously identified *USH2A* pathogenic variant [7]. Variants shown in all families are in *USH2A*. All family members were genotyped for *USH2A* deletions/duplications by MLPA. **B**. Three families were found to have the pathogenic variant *USH2A*: c.7595-2144A>G. All available family members were genotyped for this variant. Family 128 displayed an atypical phenotype. All three affected siblings were heterozygous for the variant and otherwise were genotypically identical for markers within *USH2A* (see Additional file 5). In this family the second *USH2A* pathogenic mutation is unknown, despite complete sequencing of nine Usher genes, MLPA analysis for *USH2A* deletions/duplications and massive parallel sequencing of *CIB2* and 60 genes for non-syndromic hearing loss. In addition the proband has also undergone exome sequencing and no likely disease-causing variants have been found in RP-associated genes. The two remaining families displayed typical USH2. In family 24 *USH2A*: c.7595-2144A>G segregated in trans with an already identified *USH2A* pathogenic variant [7]. Phase is unknown for family 707. Variants shown in all families are in *USH2A*.

No deletions or duplications were found in the 15 USH2 families or in the 11 atypical families with no convincing disease causing mutations. In the 23 families with a published monoallelic pathogenic/UV4/UV3 mutation in *USH2A*, seven families were identified with heterozygous deletions or duplications. In order to exclude the possibility that genetic variants beneath the MLPA oligonucleotides might be causing lack of annealing of probes to target sequences, these regions were checked by Sanger sequencing in the seven identified families for variants. In one family a heterozygous mutation was found underneath the MLPA probe binding site of the ‘deleted’ target exon, and this was considered to be a false positive result. The remaining six cases consisted of five families with heterozygous deletions, and one family with a heterozygous duplication. The genotypes of these families are shown in Table 1. Segregation analysis in each of these families showed that all deletions or duplications were *in trans* with a previously identified pathogenic variant (Figure 1A). For deleted/duplicated MLPA probe values in these families, see Additional file 3.

**Table 1:** Genotypes of probands identified with USH2A deletions/duplications and c.7595-2144A>G p.(Lys2532Thrfs*56)

**Family**	**Diagnosis**	**Gene**	**Allele 1**^**a**^	**Allele 1 predicted**	**Allele 2**^**b**^	**Allele 2 predicted**	**Pathogenicity**	**Pathogenicity**	**Ethnicity**^**c**^
				**protein change**^**a**^		**protein change**^**b**^	**allele 1**^**a**^	**allele 2**^**b**^	
46	USH2	*USH2A*	c.6862G>T	p.(Glu2288*)	**exon 40 deleted c.7452-68_7594+942del**	p.(Leu2485Thrfs*25)	Pathogenic	Pathogenic	Caucasian
148	USH2	*USH2A*	c.2299delG	p.(Glu767Serfs*21)	**exon 27 deleted c.[5299-932_5572+1023del; 5572+1100_5573-1099del]**	p.(Met1767Valfs*6)	Pathogenic	Pathogenic	Caucasian
151	USH2	*USH2A*	c.3187_3188delCA	p.(Gln1063Serfs*15)	**exon 22–23 deleted c.4628-15914_4885+472del**	**p.(Ile1544_Gly1629del)**	Pathogenic	Pathogenic	Caucasian
309	USH2	*USH2A*	c.2299delG	p.(Glu767Serfs*21)	**exon 4 deleted c.781_784+1375del**	**p.?**	Pathogenic	Pathogenic	Caucasian
657	USH2	*USH2A*	c.187C>T	p.(Arg63*)	**exon 70 deleted c.15053-26_15298-708del**	p.(Leu5019Valfs*77)	Pathogenic	Pathogenic	Caucasian
283	USH2	*USH2A*	c.2299delG	p.(Glu767Serfs*21)	**exons 4–13 duplicated**	**p.?**	Pathogenic	Pathogenic	Caucasian
24	USH2	*USH2A*	c.2299delG	p.(Glu767Serfs*21)	c.7595-2144A>G	p.(Lys2532Thrfs*56)	Pathogenic	Pathogenic	Caucasian
707	USH2	*USH2A*	c.2299delG	p.(Glu767Serfs*21)	c.7595-2144A>G	p.(Lys2532Thrfs*56)	Pathogenic	Pathogenic	Caucasian
128	Atypical Usher	*USH2A*	Unknown	Unknown	c.7595-2144A>G	p.(Lys2532Thrfs*56)	Pathogenic		

Variants in bold are novel mutations, identified in this study.

^a^ All allele 1 variants have been previously published in these individuals [7].

^b^ All allele 2 variants were identified in this study by *USH2A* MLPA or sequencing for *USH2A*: c.7595-2144A>G.

^c^ Caucasian represents UK and European.

The heterozygous duplication of exons 4 to 13 identified in family 283 is novel. To our knowledge this is the first time that a duplication of one or more exons in *USH2A* has been identified in Usher syndrome. This duplication is *in trans* with p.(Glu767Serfs*21) and segregates with disease in the family. All five identified deletions were also novel when checked against the Usher Syndrome mutation database [9]; there have been no previous reports of deletions of *USH2A* exon 4, exon 27, exon 40 and exon 70. There is one previously reported deletion of *USH2A* exons 22–23, however recent investigations have revised the deletion in that family to *USH2A* exons 22–24 [6,9]. The deletion of *USH2A* exons 22–23 identified in our study is therefore novel.

### Confirmation of *USH2A* large genomic rearrangements and breakpoint mapping

To further confirm and study the effects of the identified deletions, we isolated RNA from nasal epithelial cells from probands in two families. The proband from family 151 carried a deletion of *USH2A* exon 22–23 and p.(Gln1063Serfs*15) in a compound heterozygous state, and the proband from family 657 carried a deletion of exon 70 and p.(Arg63*) in a compound heterozygous state.

**Figure 2:**
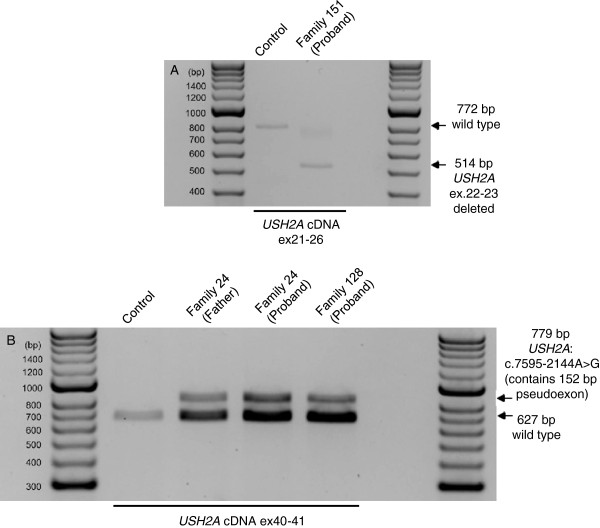
**Reverse transcription PCR confirmation of *USH2A *exon deletions and *USH2A*: c.7595-2144A>G. A**. Reverse Transcription PCR (RT-PCR) of *USH2A* exons 21–26 using RNA extracted from nasal epithelial cells as template. The proband from family 151 carries a deletion of *USH2A* exons 22–23 in trans with *USH2A*: p.(Gln1063Serfs*15). In this individual RT-PCR produced a shorter product of 514bp corresponding to a 258bp deletion of *USH2A* exons 22–23. The other allele in the person amplifies faintly. Amplification on control template produced a band of 772bp, corresponding to wild type. **B**. Reverse Transcription PCR (RT-PCR) of *USH2A* exons 40–41 using RNA extracted from fibroblasts as template. The proband and father in family 24, and the proband in family 128 all carry *USH2A*: c.7595-2144A>G. This variant is in *USH2A* intron 40, and has been previously reported to lead to the inclusion of a 152bp pseudoexon between exon 40–41 resulting in the pathogenic frameshift p.(Lys2532Thrfs*56). The proband in family 24 also carries this variant in trans with *USH2A*: p.(Glu767Serfs*21). The other disease-causing allele in family 128 could not be identified.

Reverse transcription PCR (RT-PCR) amplification between *USH2A* exons 21–26 in the cDNA of the proband from family 151 produced bands of varying intensity, shown in Figure 2A. One band of 772 bp was observed in control cDNA, and corresponded to the non-deletion allele. A smaller band of 514bp was observed in the proband from family 151 and not on control cDNA; this was the expected size for a deletion of *USH2A* exons 22–23. Multiple attempts at sequencing this band failed. The deletion of *USH2A* ex22-23 is expected to be in frame and does not appear to be subject to nonsense mediated decay. The non-deleted allele in this individual amplified very faintly, and it is suspected that this allele, *USH2A*: p.(Gln1063Serfs*15), could be subject to nonsense mediated decay or there could be preferential amplification of the short PCR product from the deletion allele.

In the proband from family 657, repeated RT-PCR attempts to amplify *USH2A* cDNA across exon 68 to 71 failed, but amplified in control nasal cDNA samples run during the same reaction. Additional RT-PCR reactions across *USH2A* exons 21–26 also amplified in controls, but not in the proband from family 657 for either *USH2A* allele. The amount of RNA used for cDNA synthesis was more than for other probands who did amplify. To assess if PCR failure was limited to *USH2A*, RT-PCR reactions for three other genes were conducted in this individual using the same template RNA sample. Amplification of two Usher genes *GPR98, WHRN*, and of the house-keeping gene *GAPDH* produced RT-PCR products of expected size in the proband from family 657. This suggests that both alleles in this individual, exon 70 deleted (which is predicted to produce the frameshift p.(Leu5019Valfs*77)) and p.(Arg63*) produce *USH2A* long isoform transcripts that are subject to nonsense mediated decay.

**Figure 3:**
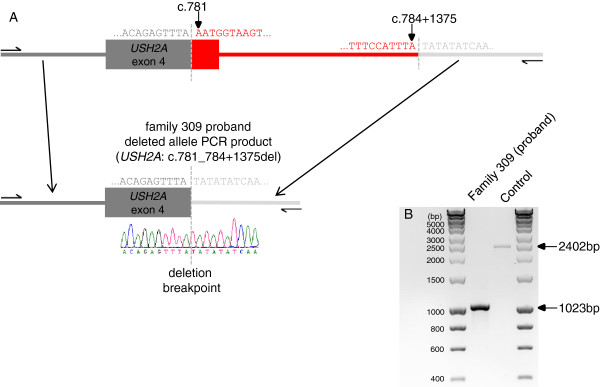
**Mapping the deletion breakpoint of *USH2A *del exon 4 in family 309. A**. Based on array CGH results PCR primers were designed to amplify across the deletion breakpoint on genomic DNA in family 309. Sequence analysis identified the exact breakpoint as *USH2A*: c.781_784+1375del. The segments in red represent the deleted regions, and grey the non-deleted. **B**. PCR products from amplification across the deletion breakpoint on genomic DNA in family 309. A smaller band, representing the deletion allele, is present in the proband from family 309 but not on control. A larger band amplifies in control, corresponding to the non-deletion allele. The proband from family 309 is heterozygous for the deletion, however, the non-deletion allele has not amplified most likely due to preferential amplification of the shorter PCR product.

A custom microarray was used to guide deletion breakpoint mapping and to confirm presence of deletions where no cDNA was available. Based on the probe coordinates, it is likely that the four single exon deletions, del E4, E27, E40 and E70 range approximately from 1.2 to 5 kb in length. The double exon deletion del E22-23 was found to be approximately 25 kb, and the duplication dup E4-13 approximately 144 kb in length. The microarray coordinates were used to design PCR primers to amplify across the genomic breakpoints of the five identified deletions. The observed PCR products in all individuals carrying deletions were smaller than expected for wild type alleles. Subsequent sequencing of the PCR products successfully identified the breakpoints for all five deletions, shown in Table 1. Interestingly the deletion of *USH2A* exon 27 in family 148 was found to comprise two deletions in close proximity (76bp apart) carried on the same allele *USH2A*: c.[5299-932_5572+1023del; 5572+1100_5573-1099del]. The deletion of exon 4 in family 309, was found to be a partial exon deletion comprising the last 4bp of exon 4 and extending into intron 4, *USH2A*: c.781_784+1375del. Deletion mapping in this family is shown in Figure 3. For all other families see Additional file 4.

### Sequencing for *USH2A*: c.7595-2144A>G p.(Lys2532Thrfs*56)

Of the 23 USH2 families with one pathogenic/UV4/UV3 mutation in *USH2A*, we identified two USH2 families with the previously reported pathogenic variant *USH2A*: c.7595-2144A>G [11]. The mutation was *in trans* with *USH2A*: p.(Glu767Serfs*21) in family 24. In family 707 phase is unknown, but an affected sibling carried the same two pathogenic mutations (Figure 1B). *USH2A*: c.7595-2144A>G was not found in the 15 USH2 families who had only UV2/UV1 variants.

Interestingly, one atypical family was also identified to carry *USH2A*: c.7595-2144A>G. This family consisted of three affected siblings, all with adult onset hearing loss (in the fourth decade), diagnosed *after* the onset of RP in the mid-teenage years. Vestibular function was normal in one sibling who has been tested. All three sisters were heterozygous for the mutation. The other disease-causing variant in this family is unknown, with no other pathogenic/UV4/UV3 mutations identified in any Usher syndrome gene. Recent analysis of *CIB2* and 60 genes causing non-syndromic hearing loss by massive parallel sequencing did not identify any other pathogenic mutations in this family. The proband has also undergone exome sequencing and no likely disease-causing variants have been found in RP-associated genes – data not shown. Genotyping of polymorphic variants in these siblings showed they share *USH2A* haplotypes, shown in Additional file 5.

### Confirmation of *USH2A*: c.7595-2144A>G on cDNA

To further study and confirm reported effects of *USH2A*: c.7595-2144A>G, we isolated dermal fibroblasts from family 24 and the atypical family 128. RT-PCR to amplify *USH2A* exons 40–41, was conducted on cDNA synthesised from these cells. All heterozygous individuals produced a band of the expected size, 627 bp, plus an additional band of 779 bp that was not observed in controls, shown in Figure 2B. Gel extraction and sequencing of the bands showed the 627 bp fragment corresponded to wild-type sequence. The sequence of the 779 bp fragment contained a 152 bp sequence from intron 40, inserted between exon 40–41. This sequence matches that previously reported by Vaché *et al.*[11] to be inserted at this point due to *USH2A*: c.7595-2144A>G. Our results confirm the findings of this previous paper regarding the splicing effects and likely pathogenicity of this deep intronic mutation.

## Discussion

It is often not possible to identify both pathogenic variants in *USH2A*[6-8]. In this study we identified eight mutations in 23 USH2 families (35%) who were known to have a monoallelic pathogenic/UV4/UV3 mutations in *USH2A* detected by Sanger sequencing. These consisted of five novel heterozygous deletions, one novel duplication in *USH2A*, and two cases with the pathogenic variant *USH2A*: c.7595-2144A>G p.(Lys2532Thrfs*56). This is the first time that a duplication in *USH2A* has been reported as a cause of Usher syndrome.

Recently the observation of monoallelic mutations in an Usher gene has been attributed to digenic inheritance, with the second mutation residing in another Usher gene [18]. Two families reported by Bonnet *et al.*[18] were identified with one pathogenic allele in *USH2A*, and one missense variant in an USH1 gene. As these changes in non-*USH2A* genes were missense variants and not definitely pathogenic alleles (nonsense, frameshifts or splice variants) they could in fact be rare benign variants, or disease modifiers. The true pathogenic mutation could be a second mutation within the *USH2A* gene which is not detected by a simple exon sequencing approach, such as those we describe here. In our 23 families with a monoallelic *USH2A* pathogenic mutation, 35% of second mutations were missed by the exon sequencing approach. Indeed, as Abu-Safieh *et al.*[19] argue in the case of Bardet-Biedl syndrome, oligogenic inheritance has not been conclusively substantiated for many diseases. Many cases of oligogenic inheritance are claimed when multiple genes are sequenced, and ethnic-specific, usually missense variants in a second gene are misinterpreted as pathogenic, as we have found in our previous study [7].

In 15 USH2 families with no pathogenic/UV4/UV3 mutations we did not find any new variants. In five of these families UV2 variants had already been identified, but treated as not likely to be disease-causing (see Additional file 1). It is possible that initial mutation classification of these variants was too strict, and these variants are more likely to be disease-causing than their initial assessment. However due to the polymorphic nature of the Usher genes, and the lack of a convincing pathogenic second mutation it is not certain if these are rare polymorphic variants or pathogenic. They have therefore been given the cautious pathogenicity ranking of UV2.

Additional explanations for missing variants in families with zero or one convincing disease-causing mutation include mutations in introns or promoters that have pathogenic effects, or mutations in an as yet unidentified rare Usher gene. Nine Usher genes were analysed in these individuals, the 10^th^ Usher gene *CIB2* was not sequenced; however this gene was documented to be a rare cause of USH1, and not the USH2 phenotype these families display. These individuals will require further investigation by exome sequencing to look for new genes, and/or sequencing of the entire genomic region or RNA analysis of known Usher genes in order to achieve a molecular diagnosis. Alternatively some may not have Usher syndrome *per se*, but could have disease caused by mutations in genes which cause non-syndromic deafness and non-syndromic RP, both of which are very genetically heterogeneous.

In 11 atypical families screened, we identified one pathogenic mutation *USH2A*: c.7595-2144A>G in family 128. Previous studies have noted pathogenic mutations in Usher genes, in patients with atypical clinical features [8,20-22]. However in the absence of a second mutation we cannot be sure that *USH2A* is definitely the cause of the atypical Usher syndrome in this family. Nevertheless, despite thorough sequencing of nine Usher genes [7], *USH2A* MLPA, and additional screening in the proband for *CIB2*, 60 genes causing non-syndromic hearing loss by massive parallel sequencing, and exome sequencing we were not able to identify any other obviously pathogenic mutations in this family. Further investigation of this family at the RNA level will be conducted in the future to help identify the missing variant.

In this study we have established that MLPA and RNA analysis can detect a significant proportion of mutations missed by exon sequencing. The nasal ciliated epithelial collection technique we use requires no anaesthetic, uses gentle brushing, lasts for a few seconds and has been very well tolerated by all participants. In addition we show for the first time that proband-derived fibroblasts can be used to study splice-affecting mutations in the clinically relevant *USH2A* long transcript. This technique has several useful implications. It is possible to transform fibroblasts into induced pluripotent stem cells, from which photoreceptors and retinal pigment epithelial cells can be derived [23]. This has recently been demonstrated with fibroblasts obtained from individuals with Usher syndrome and retinitis pigmentosa [24,25]. These cells could provide a basis for studying the biological effects of specific mutations and for testing the effects of potential new treatments currently being developed for Usher syndrome, such as small molecules or gene therapeutic approaches, in appropriate cells/cell-types [26-28]. This may help produce more translational relevant results as cells derived from patients with Usher Syndrome will have an advantage over current mouse models of USH1, which do not accurately represent the ophthalmic aspects of this disorder [29,30].

In our previous Sanger sequencing effort of nine Usher syndrome genes in 121 USH2 families, we identified two pathogenic/UV4/UV3 variants in 65% (79/121) of families, one pathogenic/UV4/UV3 variant in 21% (26/121) of families and no pathogenic/UV4/UV3 variants in 13% (16/121) of families. The *USH2A* MLPA kit we have used in this study was able to quantitatively detect changes in the copy number of an exon in a further 6 families and is therefore a useful addition in the molecular diagnosis of USH2. Taking into account all the new mutations identified and further work, these figures can be re-calculated for USH2 families. When sequenced for 9 Usher Syndrome genes, and screened for *USH2A* deletions, duplications and for the pathogenic variant *USH2A*: c.7595-2144A>G, 77% (93/121) of USH2 families have two pathogenic/UV4/UV3 mutations, 11% (13/121) have one pathogenic/UV4/UV3 mutation and 12% (15/121) have no pathogenic/UV4/UV3 variants. Considering just the *USH2A* gene, 11 families remain with mono-allelic *USH2A* mutations and a yet unidentified second mutation. A further search for intronic mutations, either by RNA analysis looking for abnormal splice variants or ‘allelic drop-out’, or high throughput sequencing of the *USH2A* genomic region, may be useful.

## Conclusions

We found that 8 of 23 (35%) mutations in individuals with a monoallelic mutation in *USH2A* can be attributed to deletions, duplications and a pathogenic deep intronic variant. Five novel deletions, one novel duplication and a previously-described intronic pathogenic mutation have been identified in this study using MLPA, sequencing and RNA analysis. Future mutation detection strategies and genetic counselling will need to take into account the prevalence of these types of mutations in order to provide a more comprehensive diagnostic service.

## Abbreviations

Array CGH: array comparative genomic hybridisation; MLPA: Multiplex ligation dependant probe amplification; RP: retinitis pigmentosa; RT-PCR: reverse transcription polymerase chain reaction; USH1: Usher Type 1; USH2: Usher Type 2; USH3: Usher Type 3; UV1: Unclassified variant 1 (likely polymorphism); UV2: Unclassified variant 2 (possibly polymorphism); UV3: Unclassified variant 3 (possibly disease-causing); UV4: Unclassified variant 4 (likely disease-causing).

## Competing interests

The authors declare that they have no competing interests.

## Authors’ contributions

HS-S completed the laboratory work, and co-wrote the manuscript with MB-G; MB-G supervised the laboratory work; EL and ARW reviewed all laboratory results and patient data and LL reviewed all audiological data; MC supervised the design of the array CGH and AFR reviewed the CGH data. LL and PLQS critically appraised the manuscript. All authors read and approved the final manuscript.

## Supplementary Material

Additional file 1 **Genotypes of all 49 families screened for *USH2A *deletions/duplications and *USH2A*: c.7595-2144A>G.** Click here for file

Additional file 2 **Clinical data from all atypical individuals and USH2 probands with *USH2A *deletions, duplications and c.7595-2144A>G.** Click here for file

Additional file 3 **MLPA probe values in families identified with *USH2A *deletion/duplications.** Click here for file

Additional file 4 **Mapping of *USH2A *deletion breakpoints on genomic DNA.** Click here for file

Additional file 5 ***USH2A *haplotype analysis of family 128.** Click here for file
