# Supplementary material for: Screening for duplications, deletions and a common intronic mutation detects 35% of second mutations in patients with USH2A monoallelic mutations on Sanger sequencing
Source: Orphanet J Rare Dis. 2013 Aug 8;8:122. doi: 10.1186/1750-1172-8-122 (PMC3751126; doi:10.1186/1750-1172-8-122)

Additional file 3. MLPA probe values in families identified with *USH2A* deletion/duplications

Deleted/duplicated exons are shown in red, normal copy number exons in green, control gene exons in blue. The green lines represent the thresholds for normal copy number (0.8 ≤ normal copy number ≤1.2). Exons with values above or below this are considered duplicated or deleted respectively. In each graph probes are ordered from right to left by PCR product size, NOT by exon number.

A. Family 46: deletion of *USH2A* exon 40 and *USH2A*: p.Glu2288* in a compound heterozygous state.
Deleted probe ratio = 0.518

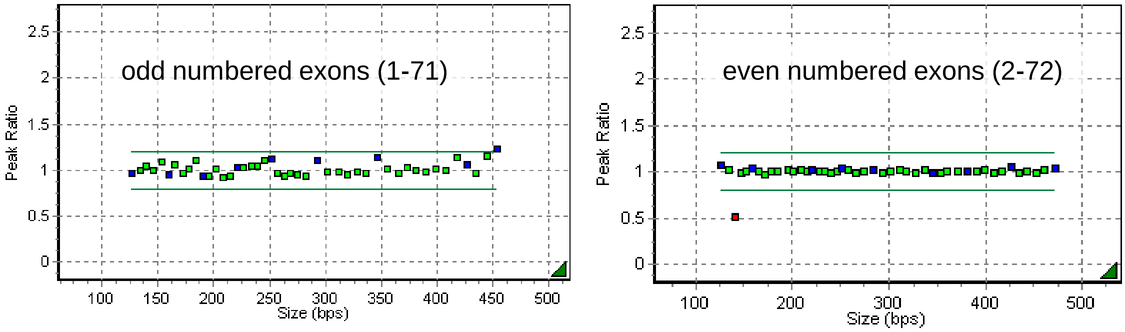

B. Family 148: deletion of *USH2A* exon 27 and *USH2A*: p.Glu767Serfs*21 in a compound heterozygous state. Deleted probe ratio = 0.538

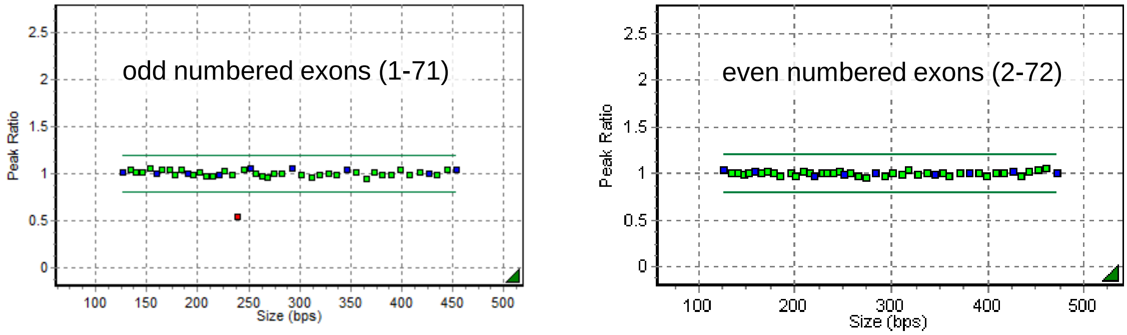

C. Family 151: deletion of *USH2A* exons 22-23 and *USH2A*: p.Gln1063Serfs*15 in a compound heterozygous state. Exon 23 deleted probe ratio = 0.516, exon 22 deleted probe ratio = 0.477

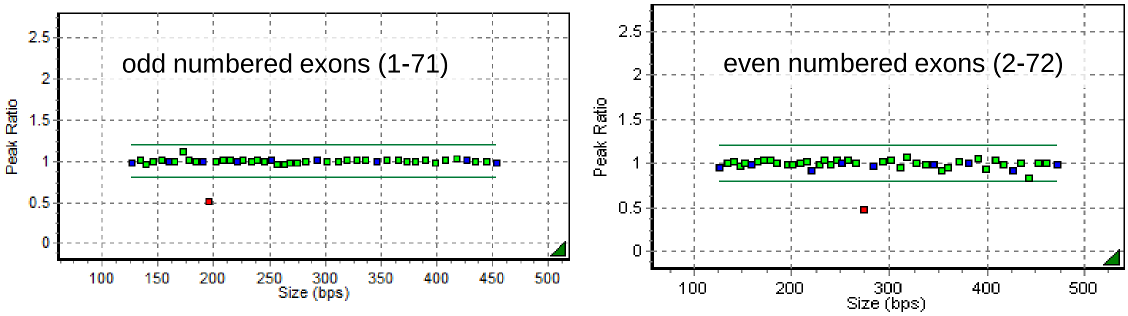

Additional file 3. MLPA probe values in families identified with USH2A deletion/duplications

(Continued)

D. Family 309: deletion of *USH2A* exon 4 and *USH2A*: p.Glu767Serfs*21 in a compound heterozygous state. Deleted probe ratio = 0.574

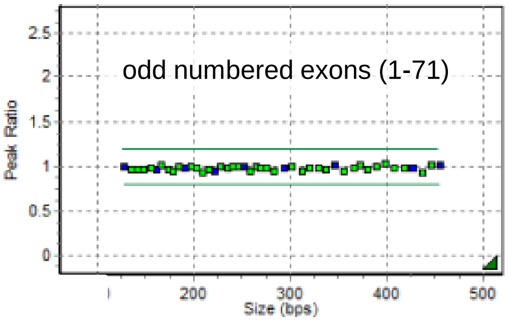

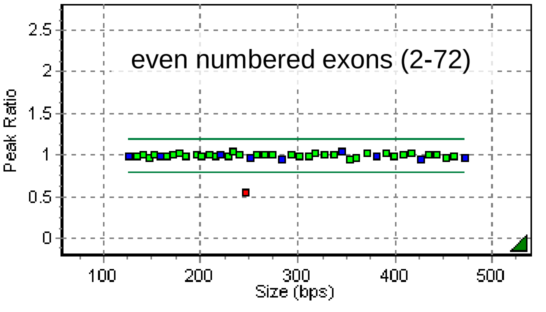

E. Family 657: deletion of *USH2A* exon 70 and *USH2A*: p.Arg63* in a compound heterozygous state. Deleted probe ratio = 0.527

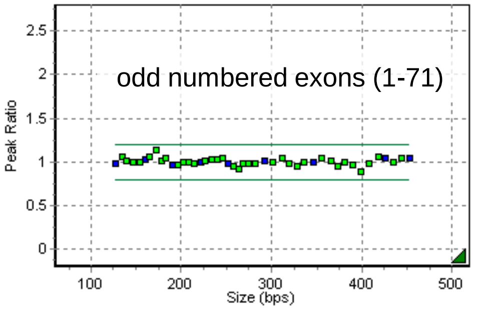

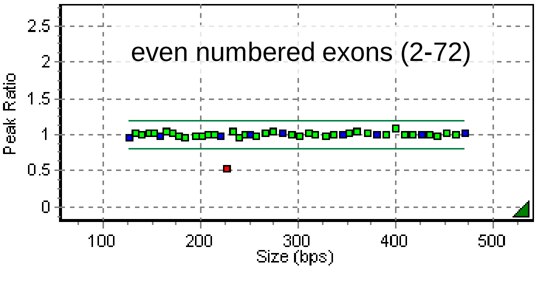

F. Family 283: duplication of *USH2A* exons 4-13 and *USH2A*: p.Glu767Serfs*21 in a compound heterozygous state.
Duplicated odd exon probe ratios from left to right: exon 5 = 1.426, exon 9 = 1.468, exon 13 = 1.431, exon 11 = 1.481, exon 7 = 1.397. Duplicated even exons from left to right: exon 4 = 1.484, exon 6 = 1.482, exon 12 = 1.443, exon 10 = 1.495, exon 8 = 1.451

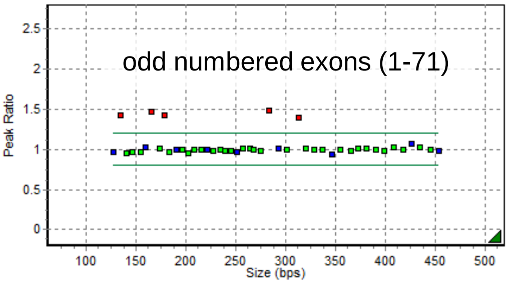

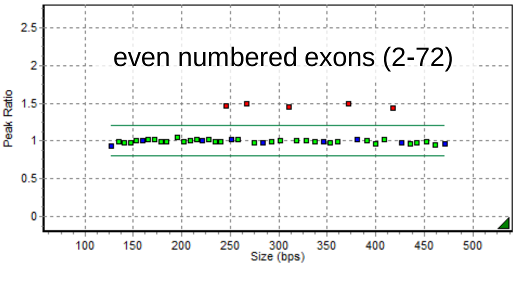

Supplement: Additional file 3 — MLPA probe values in families identified with USH2A deletion/duplications. [file 1750-1172-8-122-S3.pdf]
