# Supplementary material for: Screening for duplications, deletions and a common intronic mutation detects 35% of second mutations in patients with USH2A monoallelic mutations on Sanger sequencing
Source: Orphanet J Rare Dis. 2013 Aug 8;8:122. doi: 10.1186/1750-1172-8-122 (PMC3751126; doi:10.1186/1750-1172-8-122)
Supplement: Additional file 4 — Mapping of USH2A deletion breakpoints on genomic DNA. [file 1750-1172-8-122-S4.pdf]

Additional file 4. Mapping of *USH2A* deletion breakpoints on genomic DNA

(A-D) Based on array CGH results PCR primers were designed to amplify across the deletion breakpoints on genomic DNA, in all five families identified to carry heterozygous deletions. Diagrams of breakpoint identification are given below for four deletions, the fifth deletion is shown in figure 3. The segments in red represent the deleted regions, and grey the non-deleted regions. For *USH2A*: c.7452-68_7594+942del (*USH2A* del ex 40) the variant *USH2A*: c.7452-76T>C can be seen on the chromatogram and is carried in *cis* with the deletion allele. E. PCR across the deletion breakpoint on genomic DNA. All deletion alleles amplify in probands but not on control, producing smaller bands than wild type. Although all probands are heterozygous for deletions, for *USH2*A exon 27, exon 22-23 and exon 70 the non-deletion allele does not amplify in proband or control as it is too large. For *USH2A* exon 40 the non-deletion allele is small enough to amplify (1878bp), and is seen in both the proband from family 46 and in control.

A. *USH2A*: c.7452-68_7594+942del

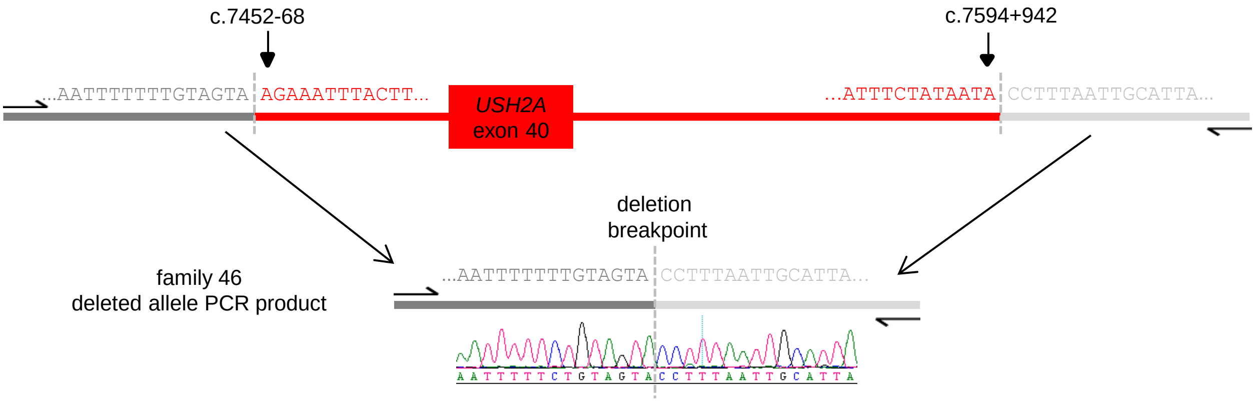

B. *USH2A*: c.[5299-932_5572+1023del; 5572+1100_5573-1099del]

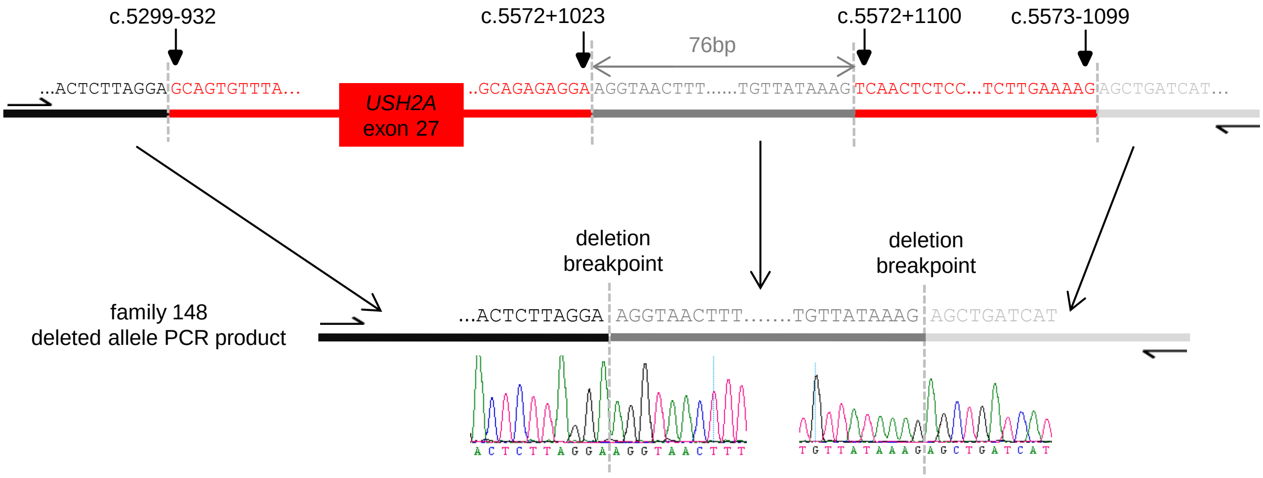

C. *USH2A*: c.4628-15914_4885+472del

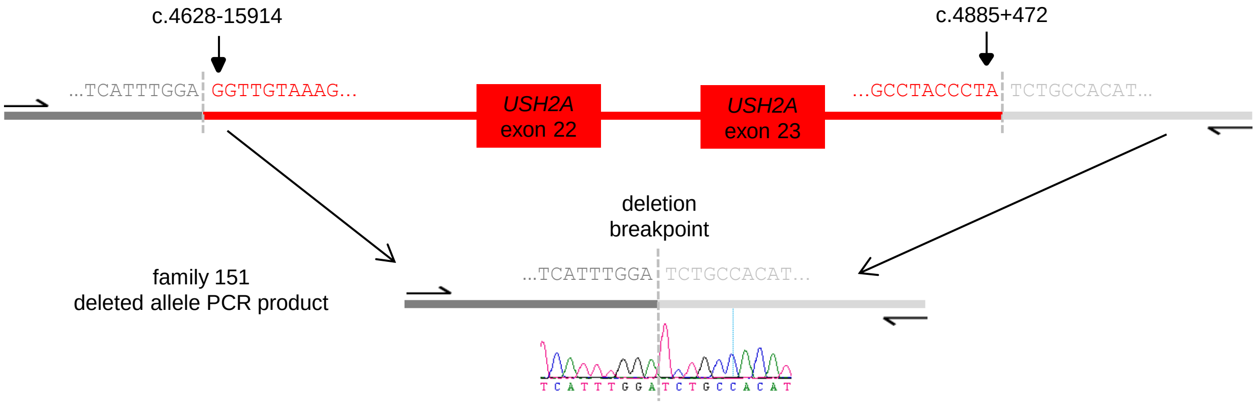

Additional file 4. Mapping of *USH2A* deletion breakpoints on genomic DNA

(continued)

D. *USH2A*: c.15053-26_15298-708del

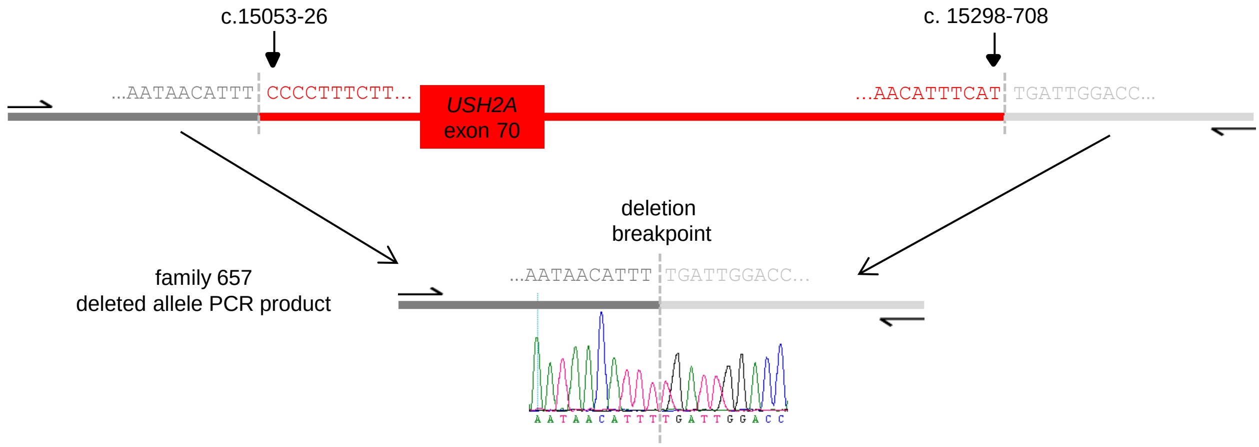

E. PCRs across the genomic breakpoints

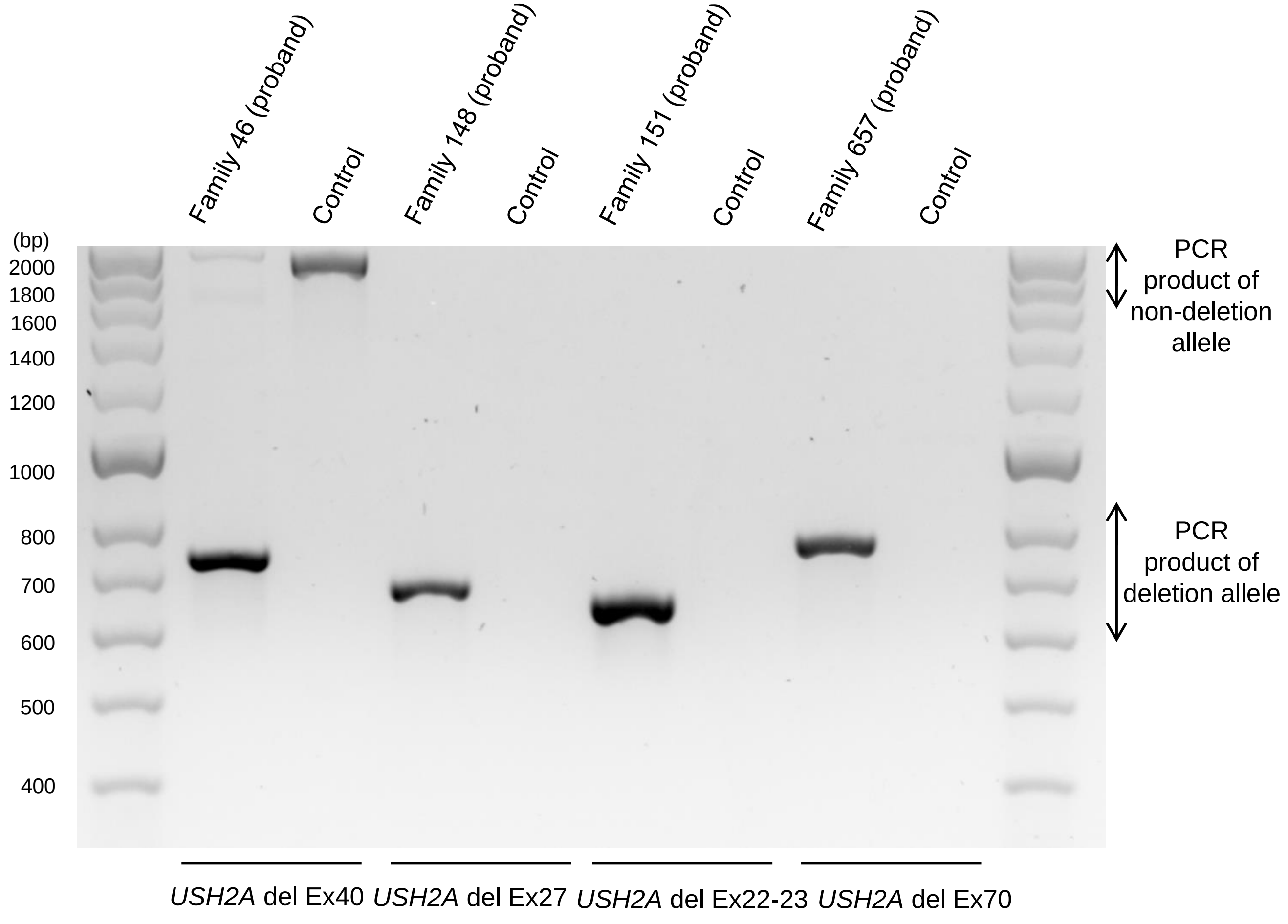

Size of deletion PCR product:	725bp	661bp	644bp	756bp
Size of wt PCR product:	1878bp	5481bp	25232bp	5743bp
