# Supplementary material for: Screening for duplications, deletions and a common intronic mutation detects 35% of second mutations in patients with USH2A monoallelic mutations on Sanger sequencing
Source: Orphanet J Rare Dis. 2013 Aug 8;8:122. doi: 10.1186/1750-1172-8-122 (PMC3751126; doi:10.1186/1750-1172-8-122)
Supplement: Additional file 5 — USH2A haplotype analysis of family 128. [file 1750-1172-8-122-S5.pptx]

## Slide 1
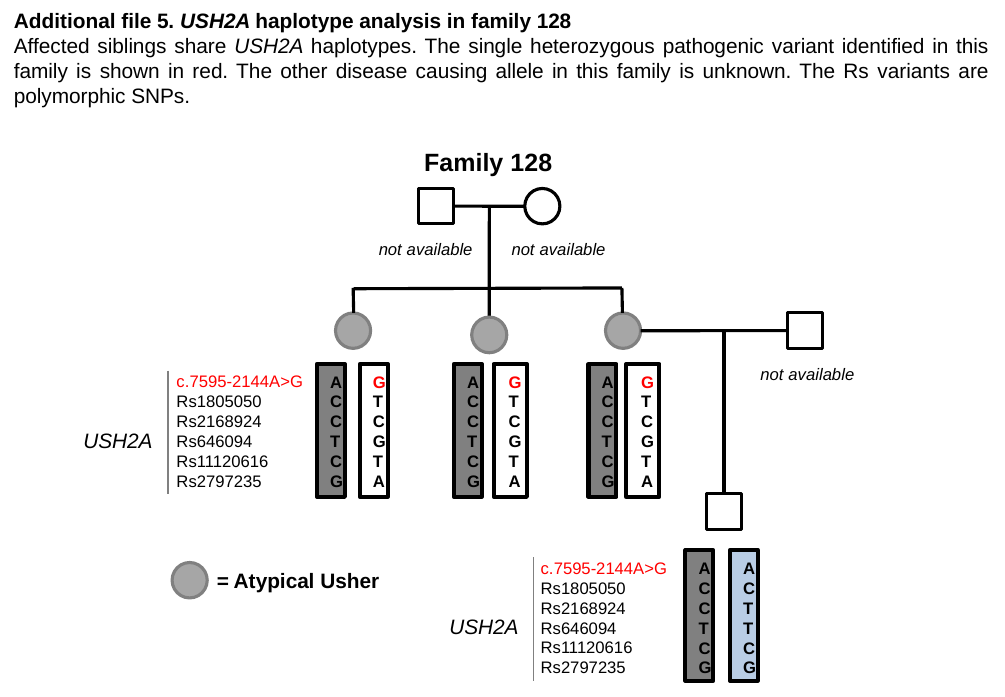

Additional file 5. USH2A haplotype analysis in family 128
Affected siblings share USH2A haplotypes. The single heterozygous pathogenic variant identified in this family is shown in red. The other disease causing allele in this family is unknown. The Rs variants are polymorphic SNPs.
Family 128
not available
not available
not available
c.7595-2144A>G
Rs1805050
Rs2168924
Rs646094
Rs11120616
Rs2797235
A
C
C
T
C
G
G
T
C
G
T
A
A
C
C
T
C
G
G
T
C
G
T
A
A
C
C
T
C
G
G
T
C
G
T
A
USH2A
c.7595-2144A>G
Rs1805050
Rs2168924
Rs646094
Rs11120616
Rs2797235
A
C
C
T
C
G
A
C
T
T
C
G
= Atypical Usher
USH2A
